# Dermoscopic, confocal, and histological analysis of cutaneous sarcoidosis

**DOI:** 10.1111/srt.13552

**Published:** 2024-01-04

**Authors:** Stephano Cedirian, Federico Venturi, Carlotta Baraldi, Emi Dika

**Affiliations:** ^1^ Oncologic Dermatology Unit IRCCS Azienda Ospedaliero‐Universitaria di Bologna Bologna Italy; ^2^ Department of Medical and Surgical Sciences (DIMEC) Alma Mater Studiorum University of Bologna Bologna Italy

**Abbreviations**
CS: cutaneous sarcoidosis
RCM: reflectance confocal microscopy

Dear Editor,

Sarcoidosis presents as a systemic granulomatous disorder with an uncertain etiology, primarily targeting lungs, intrathoracic lymph nodes and the skin.^1^ Given the heterogeneity of its manifestations, considering alternative diagnostic strategies to assess cutaneous sarcoidosis (CS) is crucial. In this case report, we present the scenario of a 60‐year‐old woman affected by an asymptomatic Chron's disease, referred to our dermatology clinic with a 6‐month history of persistent papules distributed across her back and the extensor surface of her arms. Her Crohn's disease had been effectively managed for several years, and she was not currently taking any drugs. No accompanying systemic symptoms were reported by the patient. Physical examination unveiled erythematous micropapules scattered on the back and extensor surface of her arms (Figure 1A). The lesions were mildly elevated and did not exhibit ulceration nor scaling. Dermoscopic examination showed linear and branching vessels and some areas displaying a yellowish‐orange hue (Figure 1B). Since dermoscopy was not conclusive, we proceeded with reflectance confocal microscopy (RCM) which exhibited increased vascularization and inflammatory infiltrates in the papillary dermis, alongside bright beaded‐like structures and enlarged hyperreflective cells within granulomatous structures. A surrounding bright stroma was evident as well (Figure 2). Subsequently, a 4 mm punch biopsy of one of the most recent lesions was performed. Histopathological analysis demonstrated well‐defined, non‐caseating granulomas affecting the upper and mid‐dermis. These findings were in accordance with a diagnosis of CS. The patient was treated with topical high‐potency steroids leading to the resolution the skin condition after 3 months, with a mild post‐inflammatory hyperpigmentation. CS is a granulomatous inflammatory disorder displaying different manifestations that may mimic other pathological conditions.^2^ The patient's medical history, characterized by well‐managed Crohn's disease, introduced an additional layer of intricacy to the diagnostic process. The concurrent emergence of sarcoidosis alongside Crohn's disease has been documented in a few cases in literature. Although a potential coincidental association may be implied, several areas of convergence between these two conditions exist.^3^ Indeed, both sarcoidosis and Crohn's disease are characterized by modified immune responses, notably implicating Th1 and Th17 lymphocytes, which stimulate the release of inflammatory cytokines and contributes to the formation of granulomas in both conditions. In the present case, Crohn's disease did not appear to be directly linked to the onset of CS, nor the clinical presentation suggested an extra‐intestinal manifestation of Crohn's disease. Regarding the RCM findings, increased vascularization and inflammatory infiltrates within the papillary dermis were observed. Furthermore, distinctive beaded‐like structures and enlarged hyperreflective cells were identified within granulomatous formations, accompanied by a surrounding bright stroma. These observations align with prior case reports that have documented similar RCM patterns in CS.^4^ Due to CS deceptive clinical presentation, it is crucial to emphasize that subepidermal granulomas, a pivotal histological finding, can be visualized through RCM which provides more substantial histological insights by precisely localizing and targeting the region of interest prior to undertaking a biopsy.^5^ Moreover, due to the potential correlation of CS with systemic involvement and tumorigenesis, its early identification is key to better patient management.

**FIGURE 1:**
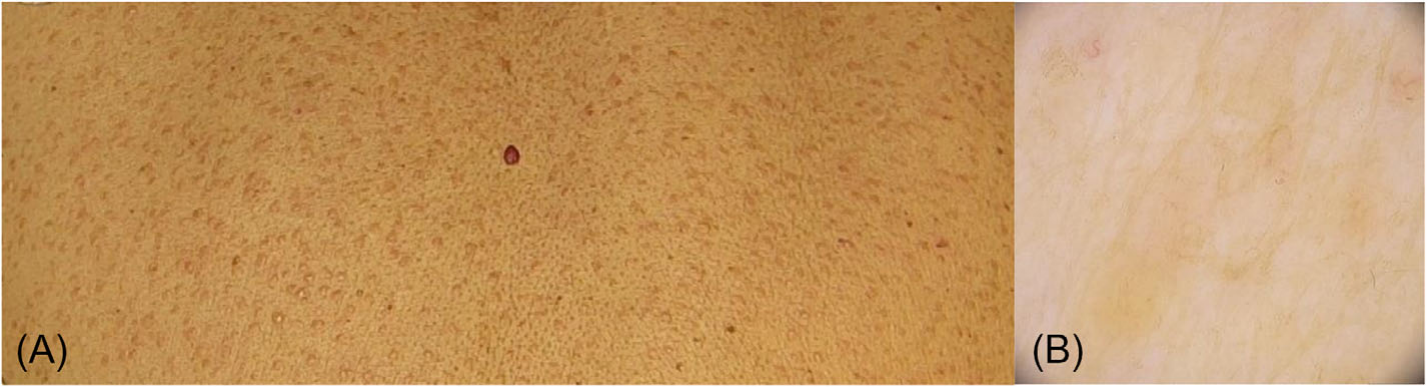
Clinical presentation of the multiple erythematous itchy micropapules scattered on the back and extensor surface of the arms of a 60‐years old patient with a history of Crohn's disease (A). Dermoscopic examination revealed linear and branching vessels and some areas displaying a yellowish‐orange hue (B).

**FIGURE 2:**
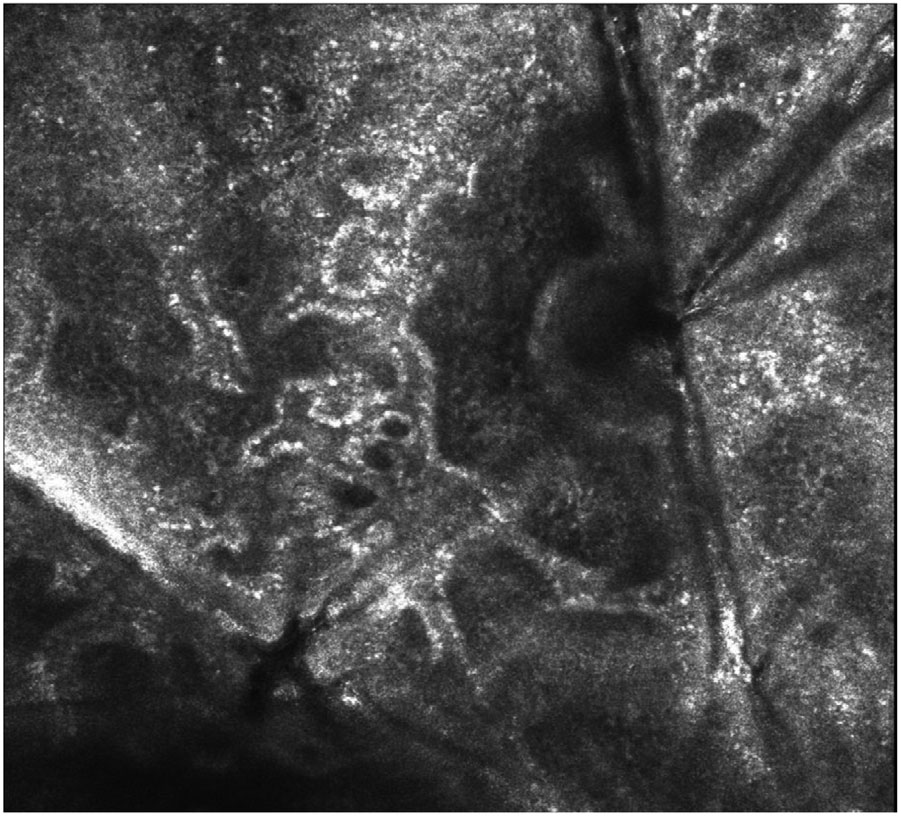
RCM analysis performed on a scattered erythematous papule of the right forearm exhibits increased vascularization and inflammatory infiltrates in the papillary dermis, alongside bright beaded‐like structures (orange arrow) and enlarged hyperreflective cells (green arrow) within granulomatous structures. A surrounding bright stroma was visible as well. RCM, reflectance confocal microscopy.

## CONFLICT OF INTEREST STATEMENT

The authors have no conflict of interest to declare. The patient in this manuscript have given written informed consent to publication of her case details.

## Data Availability

Data sharing is not applicable to this article as no new data were created or analyzed in this study.
